# MCPH1, mutated in primary microcephaly, is required for efficient chromosome alignment during mitosis

**DOI:** 10.1038/s41598-017-12793-7

**Published:** 2017-10-12

**Authors:** M. Arroyo, R. Kuriyama, M. Trimborn, D. Keifenheim, A. Cañuelo, A. Sánchez, D. J. Clarke, J. A. Marchal

**Affiliations:** 1 Departamento de Biología Experimental, Universidad de Jaén, Jaen, Spain; 2 Department of Genetics, Cell Biology and Development, University of Minnesota, Minneapolis, USA; 3 Institute of Medical Genetics and Human Genetics, Charite Virchow-Klinikum Hospital, Berlin, Germany

## Abstract

MCPH1 gene, mutated in primary microcephaly, regulates cell progression into mitosis. While this role has been extensively investigated in the context of DNA damage, its function during unperturbed cell cycles has been given less attention. Here we have analyzed the dynamics of chromosome condensation and cell cycle progression in MCPH1 deficient cells under undamaging conditions. Our study demonstrates that chromosome condensation is uncoupled from cell cycle progression when MCPH1 function is lacking, resulting in cells that prematurely condense their chromosomes during mid G2-phase and delay decondensation at the completion of mitosis. However, mitosis onset occurs on schedule in MCPH1 deficient cells. We also revealed active Cdk1 to be mandatory for the premature onset of chromosome condensation during G2 and the maintenance of the condensed state thereafter. Interestingly, a novel cellular phenotype was observed while monitoring cell cycle progression in cells lacking MCPH1 function. Specifically, completion of chromosome alignment at the metaphase plate was significantly delayed. This deficiency reveals that MCPH1 is required for efficient chromosome biorientation during mitosis.

## Introduction

MCPH1 primary microcephaly (OMIM 608585) is a rare human syndrome that results in pronounced reduction of the cerebral cortex, mental retardation and delayed growth^1,2^. While the clinical phenotype is identical to the other genetic variants of MCPH syndrome (MCPH1-MCPH14) described so far^3–5^, from a cellular perspective MCPH1 syndrome revealed a unique altered pattern of chromosome condensation. Routine cytogenetic analysis in MCPH1 patients first reported an increased frequency of cells with condensed chromatin with an intact nuclear envelope, named “prophase-like cells” (PLCs)^6–9^. PLCs are observed due to both premature onset of chromosome condensation in G2-phase and delayed decondensation in early G1 cells following nuclear division^6,7^. Chromosome condensation at these inappropriate cell cycle stages has also been observed in human cells transiently depleted of MCPH1 by siRNAs and in Mcph1−/− mouse models^10,12–14^. This phenotype is therefore considered a cellular hallmark of MCPH1 deficiency.

Mechanistically, MCPH1-related premature chromosome condensation is a result of the premature loading of condensin II onto the chromatin during G2^14,15^. Cell-free assays demonstrated that MCPH1 associates with chromatin through its N-terminal domain at the same binding sites as condensin II, thus inhibiting the loading of the condensin II complex^15^. Other studies have provided indirect evidence that unscheduled activation of Cdk1 kinase directly contributes to the premature onset of chromosome condensation. In MCPH1 mutant cells released from early S-phase synchrony, the levels of inactive Cdk1, phosphorylated at tyrosine 15 (PY15-Cdk1), become drastically reduced as soon as 4 h after release. This correlates temporally with the onset of premature condensation^16,17^. Other data indicate that premature activation of Cdk1 in MCPH1 syndrome relies on inappropriately high levels of active Cdc25A^16,18^. Since Cdc25 activation is normally regulated by the checkpoint kinases Chk1 and ATR, the data potentially place the Cdc25-Chk1-ATR pathway under MCPH1 control^16,18^.

MCPH1 is a multi-functional protein with proposed roles in telomere maintenance, DNA repair, centrosome function and tumor suppression^19^. While a large collection of studies have delineated the role of MCPH1 during cell cycle progression under conditions where DNA is damaged, its function during unperturbed cell division has seen less attention. In relation to this, some studies suggest that MCPH1 deficiency leads to premature entrance into mitosis^17,18^. This conclusion was mainly supported by the increased frequency of H3PS10 positive cells observed in either siRNA-MCPH1 treated cells or patient cell cultures. However, no studies have carefully measured the timing of mitosis and cell cycle transitions in cells with deficient MCPH1. Therefore, it is currently unknown whether the defect lies exclusively in the regulation of chromosome condensation or whether other key events of mitotic progression are also altered.

In the present work we have tracked in real time the dynamics of chromosome condensation and cell cycle progression in MCPH1 deficient cells during unperturbed cell division cycles. This analysis revealed that cells without MCPH1 prematurely condense their chromosomes during mid G2-phase and decondense them subject to a delay at the completion of mitosis. However the onset of mitosis, based on nuclear levels of mitotic markers and the timing of nuclear envelope breakdown, occurs on schedule in MCPH1 deficient cells. We also provide evidence that active Cdk1 is mandatory for the premature onset of chromosome condensation in MCPH1 syndrome. Interestingly, our analysis demonstrates that, in addition to regulating the timing of chromosome condensation, MCPH1 is also required for efficient chromosome alignment during prometaphase.

## Results

### Tracking PLC dynamics and mitosis progression in cells lacking MCPH1 function

**Figure 1:**
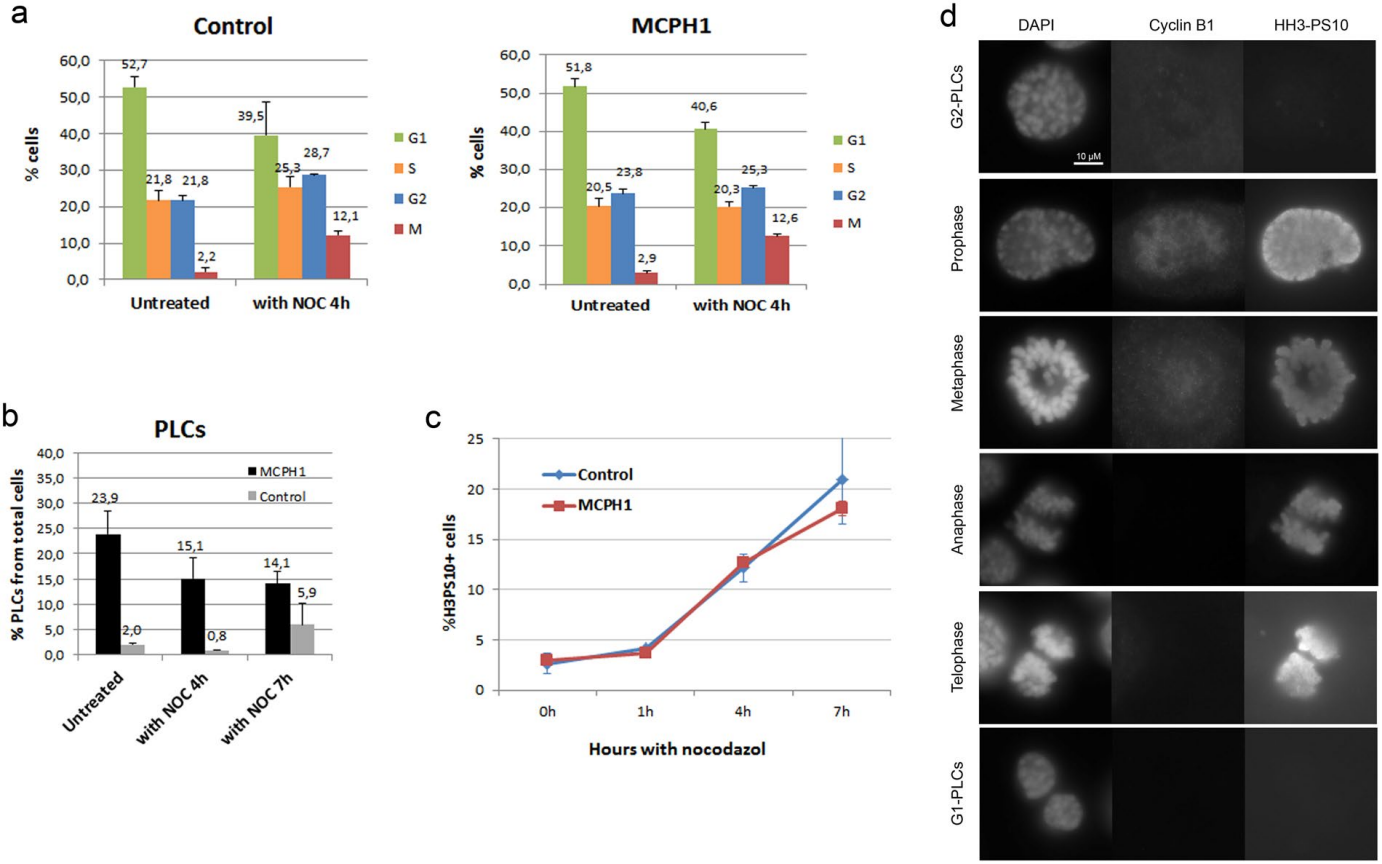
Cell cycle progression and dynamics of prophase like-condensation in control and MCPH1 patient cells. (**a**) Graphs comparing the cell cycle distribution, determined by FACS analyses, in untreated cell samples or cell samples incubated with nocodazole for 4 hours to induce M arrest. (**b**) For each sample from A, we determined in parallel the fraction of PLCs (prophase-like cells) by microscopic analyses of cytogenetic preparations. We also included cells incubated with nocodazole for 7 hours in these analyses. More than 500 cells were scored per sample. As expected, PLC frequency was negligible in control cells. Mean and S.D. data from at least 3 independent experiments are shown. Mean values are indicated. (**c**) Rate of H3PS10 positive cells in control and MCPH1 patient cells, determined by FACS after incubation with nocodazole for the indicated time points. Data from two independent experiments are presented. (**d**) Immunolocalization using antibodies against Cyclin B and Histone H3-PS10 proteins in proliferating cells from one MCPH1 patient. PLCs (“Prophase-like cells”) refers to cells with condensed chromatin inside a retained nuclear envelope, either as a consequence of premature onset of chromosome condensation or delayed decondensation at the end of mitosis. Note that G2-PLCs and real prophases are indistinguishable by DAPI staining but showed different IF patterns for both markers. G1-PLCs are also negative for both markers. Only a minor fraction of the observed cells with the typical morphology of prophase cells contained nuclear signals for both Cyclin B and Histone H3-PS10 proteins.

We first determined the frequency of “Prophase-like cells” (PLCs) in log-phase cultures of MCPH1 patient lymphoblasts, identified through cytomorphological analysis (Fig. 1a and b). In parallel we determined the mitotic index by FACS analysis of mitotic markers (phosphorylation of histone H3). The FACS data revealed that only 2.9% (±0.5%) of MCPH1 patient cells were histone H3PS10 positive (Fig. 1a), while within the same sample 23.9% (±4.5%) of the cells were PLCs (Fig. 1b). Therefore, most PLCs could not have been mitotic cells. In order to gain direct evidence that this is the case, we used immunofluorescence staining to simultaneously observe histone H3PS10, cyclin B and chromosome morphology (Fig. 1d). This analysis revealed only a minor fraction of total PLCs (11%) that were positive for both markers and could, therefore, be defined as mitotic cells. Both markers showed the expected staining patterns in cells that were in prometaphase or beyond (Fig. 1d). Similar results were obtained when we observed histone H3PS28 and cyclin B proteins (Supplementary Figure 1). Interestingly, the fraction of H3PS10 positive cells was slightly although not significantly increased in MCPH1 patient cells (2.9 ± 0.5%) compared with control cells (2.2 ± 1.1%), consistent with previous reports^16,18^. Finally, we compared the dynamics of mitotic entry in both control and MCPH1 patient cells by determining the frequency of H3PS10 positive cells versus time after adding the spindle poison nocodazole to the cultures (Fig. 1c). This revealed that mitotic cells accumulate at similar rates in both samples, suggesting no significant differences in the rate of progression into mitosis in control and MCPH1 patient cells.

Since PLCs are evidently a consequence of premature condensation in G2 and delayed decondensation at the completion of mitosis^6^, we also compared their frequency within the G2 and G1 cell populations. As both – G1 and G2 PLCs - are indistinguishable by morphological analysis, we compared the PLC frequency before and after incubation with nocodazole. During this incubation G1 PLCs that decondense their chromosomes are not replaced by new G1 PLCs because progression through mitosis is blocked. Consequently, only G2 PLCs will remain in the population. In untreated patient cells, we observed 23.9% (±4.5%) of the population were PLCs, . After nocodazole incubation for 4 hours this frequency was reduced to 15.1% (±4.2%), and a similar frequency was observed when the incubation was prolonged for 7 hours (14.1 ± 2.5%) (Fig. 1b). In control cells the PLC frequency remained negligible as expected. Thus, we estimate that, in cycling cells from MCPH1 patients, approximately 15% and 9% of total cells are G2 PLCs versus G1 PLCs respectively (Fig. 1b). These data are in agreement with live-cell microscopy studies (see below) and with previous estimations^14^. The FACS analysis showed that G2 and G1 populations represent 23.8% (±1.0%) and 51.8% ( ± 2.0%) of total cells within the same untreated patient samples (Fig. 1a). Accordingly, we assume that in the MCPH1 patient cells, premature chromosome condensation occurs at mid-G2 phase, as previously proposed^16^, while decondensation is delayed at the completion of mitosis, covering approximately 20% of the subsequent G1 phase.

**Figure 2:**
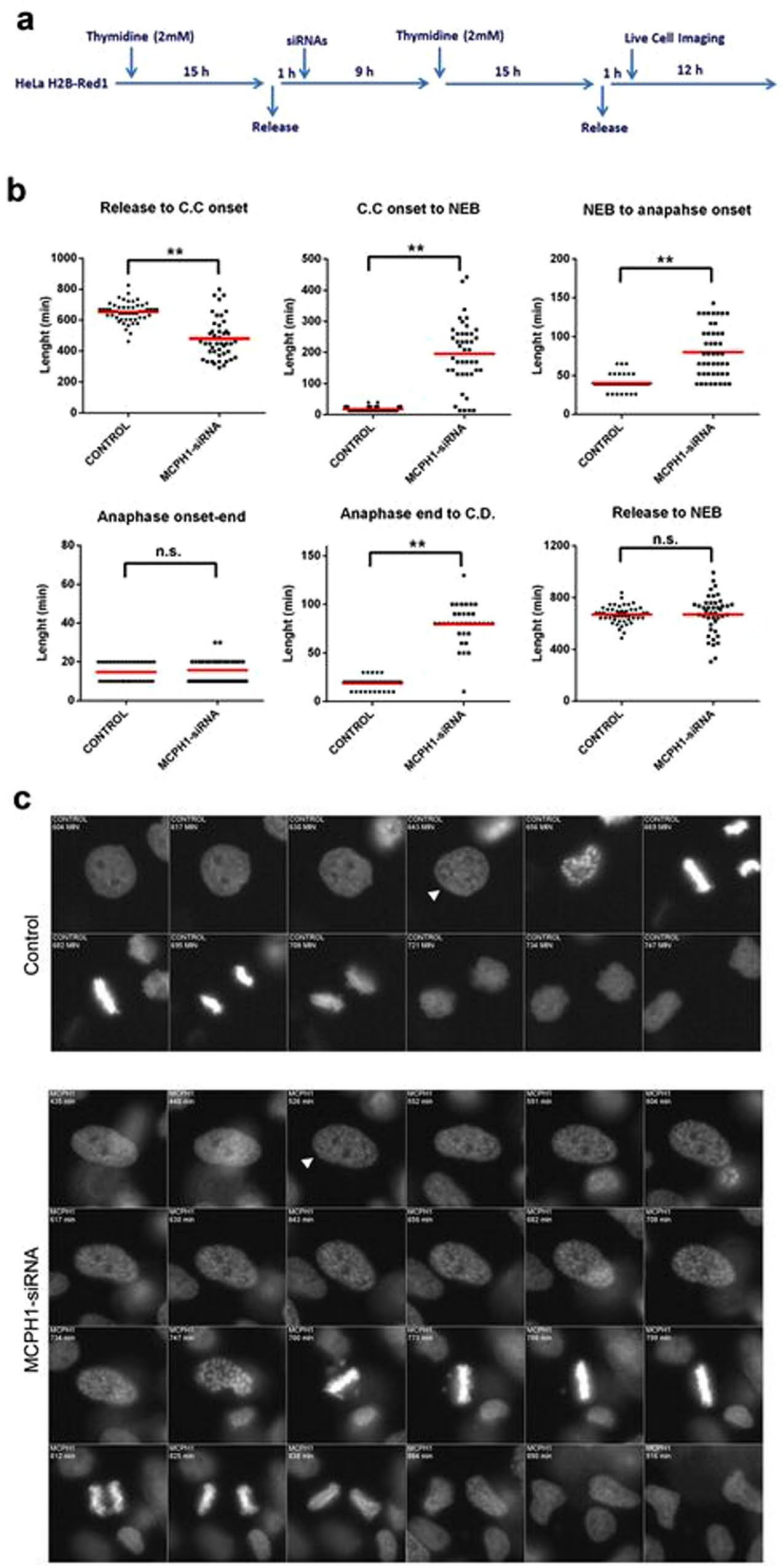
Analyses of mitosis progression by live-cell microscopy in cells depleted of MCPH1 function by siRNAs. (**a**) A brief description of the experimental procedure: HeLa cells stably expressing fluorescent histone H2B fused to Red1 were synchronized at the G1/S border by double thymidine block. MCPH1 depletion was achieved by transfection with siRNAs (oligo siRNA-MCPH1-3) during the release from the first thymidine block. Time-lapse images were collected using “Nikon Biostation IM Cell incubator” one hour after the release from the second thymidine block. (**b**) Dot-plots showing the time interval between different key mitotic events in minutes for untreated and MCPH1 depleted cells. The red line indicates the mean value. C.C. = chromosome condensation; NEB = nuclear envelope breakdown; C.D. = chromosome decondensation. At least 40 cells were analyzed in each case. Statistical comparisons for the mean and median data were done by T-student and Wilcoxon (W) tests respectively. **p < 0.01; N.S. not significant. (**c**) Selected frames showing the mitosis progression of representative HeLa H2B/Red1 cells from both untreated and MCPH1-depleted cell samples. Time from release is indicated in minutes. Arrow heads point to the first time point when chromosome condensation is clearly observable after release. Full movies are included in videos 1 and 2.

To analyze the dynamics of chromosome condensation and progression through mitosis in individual cells lacking MCPH1 function we performed live-cell fluorescence microscopy using HeLa cells stably expressing histone H2B-Red1. We employed double-thymidine synchronization to arrest cells at the G1/S border then analyzed progression through the subsequent S-phase, G2 and mitosis. MCPH1 depletion was achieved by siRNA transfection using validated siRNA oligos which knocked-down the MCPH1 protein levels efficiently^11^ (experimental procedure outlined in Fig. 2a; Supplementary Figure 3). We also confirmed that the cellular phenotypes arising after MCPH1 depletion, that is, the appearance of PLCs and altered chromosome structure in mitosis^6,7,10^ were both recapitulated using this protocol (Supplementary Figure 2). Results from live-cell analyses are presented in Fig. 2, and videos 1 and 2 (Supplementary information). We observed that cells depleted of MCPH1 condense their chromosomes 479 (±132) minutes after release from thymidine arrest, and that in these cells the PLC phenotype (with evident chromosome condensation) persisted for an average of 195 (±132) minutes. In control cells chromosome condensation was first evident much later (651 ± 63 minutes after release), and occurred not long (17 ± 7 minutes) before NEB (nuclear envelope breakdown) as expected. Importantly, NEB occurred on schedule in MCPH1 depleted cells, at the same time as in control cells (671 ± 149 minutes in MCPH1, 668 ± 64 minutes in control; time after release). Strikingly, MCPH1 depleted cells required substantially more time to progress from late prophase to the onset of anaphase: the interval from NEB until anaphase onset was 40 ± 9 minutes in control cells and 80 ± 33 minutes in MCPH1 depleted cells. Finally, we determined the timing of progression through late mitosis. While the duration of anaphase was not altered in MCPH1 depleted cells (16 ± 6 minutes) compared with controls (15 ± 5 minutes), the time that cells remained with condensed chromatin once chromosome segregation had been completed was longer. MCPH1 depleted cells required 80 (±20) minutes to complete this last step while control cells required only 19 (±6) minutes. When these analyses were repeated using a second non-overlapping siRNA similar results were obtained (Supplementary Figure 4).

In summary, the data demonstrate that when MCPH1 is depleted, cells condense their chromatin prematurely, in mid-G2 phase, and remain in that state (termed PLCs) for 195 minutes before initiating NEB. Condensation persists for an additional 80 minutes once chromosomes have segregated, covering part of the subsequent G1 phase. Despite the altered condensation dynamics, NEB occurs on time in MCPH1 depleted cells compared with controls. Condensation is therefore dramatically uncoupled from cell cycle progression. Importantly, similar results were observed when these analyses were repeated using a different isolate of HeLa stably expressing histone H2B-GFP, and also in Hct-116 H2B-GFP cells, a human modified colorectal carcinoma cell line (Supplementary Figure 5; video 8 at supplementary information).

### MCPH1 is required for efficient chromosome biorientation in prometaphase

**Figure 3:**
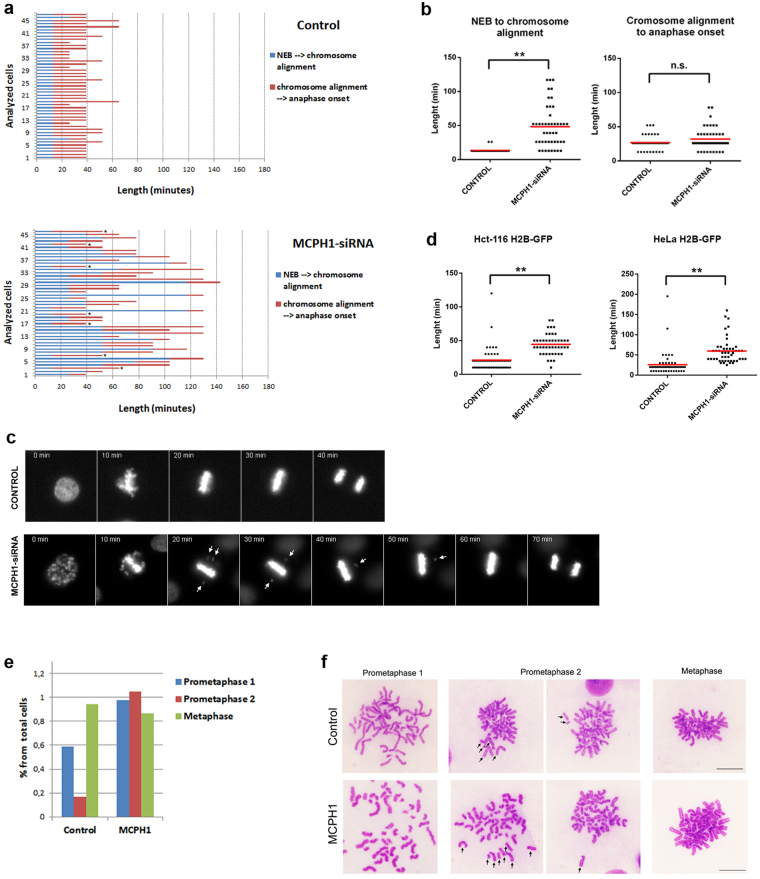
Analyses of prometaphase and metaphase progression in MCPH1 cells lacking MCPH1 function. (**a**) Live-cell microscopy analyses in HeLa H2B-Red1 cells depleted of MCPH1 by siRNAs (oligo siRNA-MCPH1-3). Graphs show the time required in individual control or MCPH1-depleted cells to i) align all the chromosomes at the metaphase plate (blue), ii) initiate chromosome segregation after metaphase alignment (red). Cells marked with an asterisk did not show the PLC phenotype before NEB. (**b**) Dot-plots from the data in A. The red line indicates the mean value. At least 40 cells were analyzed in each case. Statistical comparisons for the mean and median data were done by T-student and Wilcoxon (W) tests respectively. **p < 0.01; N.S. not significant. (**c**) Representative cells showing the progression through prometaphase and metaphase from both HeLa untreated and MCPH1-siRNA treated cell samples. Time from nuclear envelope breakdown (first frame) is indicated in minutes. Note that in the MCPH1-depleted cell most chromosomes align at the metaphase plate on time but some require more time to complete the process (pointed by arrows). Once metaphase alignment is fully achieved, cell progresses into anaphase without delay. (**d**) Dot-plots showing the duration of prometaphase in Hct-116 H2B-GFP and HeLa H2B-GFP cells depleted of MCPH1 by siRNAs. Analyses performed as described in **a** and **b**. (**e**) Percent of the corresponding mitotic stages observed in control and MCPH1 patient lymphoblasts. Analyses were performed by microscopic inspection of cytogenetic preparations obtained following a protocol that preserves the organization of chromosomes on the mitotic spindle^12^. At least 100 mitotic cells were counted and classified according to the alignment stage of their chromosomes as follows: Prometaphase 1, if no signs of metaphase plate conformation is observed; Prometaphase 2, if most chromosomes are already aligned into a plate but few of them remain far; Metaphase, when all chromosomes are finally aligned (representative pictures in **f**; arrows point to chromosomes that remain far from the already formed plate). For better interpretation of our results, the data were adjusted considering the total amount of cells that were in either prometaphase or metaphase in control (1.7%) and patient (2.9%) cells (n = 1000). Representative data from analyses performed by two different persons are presented.

The extended interval between NEB and anaphase onset in MCPH1 depleted cells prompted a closer examination of prometaphase (and metaphase) by live-cell analysis (Fig. 3a–c). This revealed that MCPH1 depleted cells require more time to align all the chromosomes at the metaphase plate compared with controls (14 ± 3 minutes in control, 48 ± 30 minutes in MCPH1). As depicted in Fig. 3c, most chromosomes in MCPH1 depleted cells typically became aligned at the metaphase plate by the first frame after NEB (a time interval of approximately 10 minutes). Despite this, a small number of unaligned chromosomes usually persisted and required more time to complete biorientation. Once full alignment was achieved, cells progressed into anaphase without significant delay (27 ± 10 minutes in control, 32 ± 16 minutes in MCPH1). Similar results were obtained when these analyses were repeated using a second non-overlapping siRNA (Supplementary Figure 4). The observed prolongation of prometaphase was also recapitulated in another HeLa cell line stably expressing Histone H2B-GFP, and also in Hct-116 H2B-GFP cells (Fig. 3d). Importantly, as we could track mitosis and chromosome condensation in individual cells, we observed that only those cells displaying a clear PLC phenotype before NEB (i.e. indicating efficient MCPH1 depletion), then had an extended prometaphase duration (Fig. 3a). On the other hand, rare cells within the MCPH1-siRNA treated population where premature condensation did not occur (i.e. indicative of unsuccessful MCPH1 depletion), did not delay in prometaphase.

In order to confirm these findings that were based on siRNA-mediated loss of MCPH1 function, we next investigated if a similar alteration could be observed during prometaphase in MCPH1 patient cells. This analysis was conducted through detailed microscopic inspection of mitotic preparations obtained from lymphoblast cell cultures following a protocol that preserves the organization of chromosomes on the mitotic spindle^12^ (Fig. 3e and f). In both control and patient samples, mitotic cells were counted and scored according to the alignment stage of their chromosomes as follows: prometaphase 1, if no sign of the metaphase plate conformation was observed; prometaphase 2, if most chromosomes were aligned on the metaphase plate but few chromosomes remained away from the plate; and metaphase, where all chromosomes were aligned at the metaphase plate (representative pictures in Fig. 3f). The data obtained are consistent with the process of chromosome alignment taking longer in patient cells compared with controls. While in the MCPH1 patient sample 0.97% and 1.05% of total cells where classified as prometaphase 1 and 2 respectively, in the control sample 0.58% of total cells were in prometaphase 1 and only 0.17% of cells were classified as prometaphase 2. The fraction of cells in metaphase, however, was similar in both samples (0.87% and 0.94% of total cells in control and patient respectively). Remarkably, cells classified as prometaphase 2 and frequently observed in patient cells - and only rarely in controls-, resemble those with an established plate but a few unaligned chromosomes observed by live-cell imaging in MCPH1 depleted HeLa cells (compare Fig. 3c and f). When these analyses were repeated in U2OS cells depleted of MCPH1 function by siRNA, similar results were observed (Supplementary Figure 6). Overall, our data provide clear evidence that MCPH1 is required for efficient chromosome alignment during mitosis, a novel cellular phenotype related to the lack of function of MCPH1.

In the live-cell experiments we did not detect significantly increased frequencies of apoptotic cells or multipolar mitosis in cells depleted of MCPH1 compared with controls. However, we did observe an increased occurrence of anaphase errors including bridged or lagging chromosomes in anaphase (Supplementary Figure 7). Most mis-segregated chromosomes gave rise to micronuclei (data not shown). Although the basal level of these errors clearly differed between the different cell lines that were analyzed, the frequency of anaphase errors was found always to be higher after MCPH1 depletion.

### Premature chromosome condensation in MCPH1 syndrome requires active Cdk1

**Figure 4:**
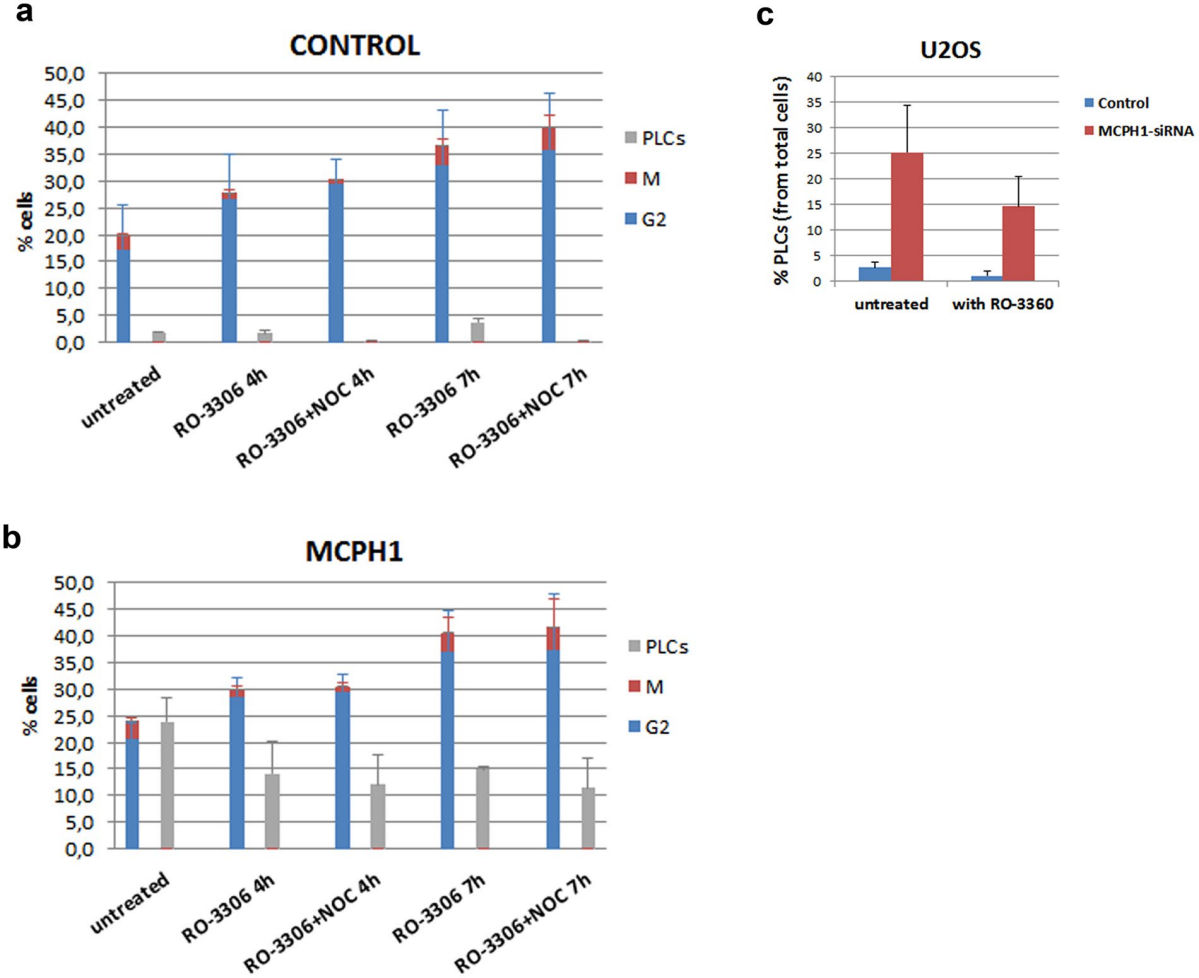
Analyses of cell cycle progression and dynamics of chromosome condensation in control and MCPH1 deficient cells treated with the Cdk1 inhibitor RO-3306. Fraction of M and G2 cells, determined by FACS analyses, in control (**a**) and MCPH1 patient cells (**b**) after either 4 h or 7 h of incubation with RO-3306 alone or combined with nocodazole (NOC), a spindle poison that causes M arrest. For each sample, we determined in parallel the fraction of PLCs by microscopic analyses of cytogenetic preparations. These data confirm that cells do not escape from G2 arrest. The fraction of PLCs is constantly reduced after prolonged incubation with RO-3306 in patient cells. In control cells, as expected, PLCs are nearly abseny. (**c**) Determination of the PLC frequency in U2OS cells depleted of MCPH1 by siRNAs and incubated with the Cdk1 inhibitor RO-3360 for 6 h. Mean and SD data are shown in all cases. Reduction of PLCs rate after RO-3306 treatment in b and c was statistically significant (p < 0.01, Χ^2^ test).

It has been proposed that premature onset of chromosome condensation depends on premature activation of Cdk1 during G2 in MCPH1 deficient cells^16,17^. To test this directly, we examined MCPH1 deficient cells treated with RO-3306, a small-molecule inhibitor of Cdk1 that reversibly arrests human cells at the G2/M border)^20^. We compared the fractions of G2, mitosis (M) and PLCs in control and MCPH1 patient lymphoblast cells incubated with RO-3306 for 4 h and 7 h (Fig. 4). The data reveals that during prolonged incubation with the Cdk1 inhibitor there is a progressive accumulation of G2 cells in both control and MCPH1 cells (Fig. 4a and b). The G2 arrest was confirmed by simultaneous incubation with RO-3306 and nocodazole. In this case, we observed a progressive accumulation of G2 cells but almost no accumulation of mitotic cells despite the treatment with the spindle poison (Fig. 4a and b). Interestingly, in parallel, the PLC frequency progressively diminished in MCPH1 patient cells, both with either RO-3306 alone or in combination with nocodazole (Fig. 4a and b). In control cells, PLCs were rarely observed, as expected. A similar reduction in the PLC frequency was observed in U2OS cells depleted of MCPH1 function by siRNAs then treated with RO-3306 (Fig. 4c).

We next monitored cell cycle progression and the dynamics of chromosome condensation after Cdk1 inhibition by live-cell fluorescence microscopy in HeLa H2B-Red1 cells. Cells were synchronized at G1/S by double-thymidine block and MCPH1 depletion was achieved by transfection with siRNAs. Immediately after release from the second thymidine arrest, RO-3306 was added to the cultures and time-lapse microscopy was performed (videos 3 and 4, supplementary information). This analysis revealed that neither control nor MCPH1 depleted cells were able to enter mitosis during the 20 hours that were recorded following release from the synchrony. Importantly, MCPH1-depleted cells did not undergo chromosome condensation during that time, i.e. no PLCs were observed. Therefore, the onset of premature chromosome condensation during G2 in cells lacking MCPH1 requires active Cdk1. We performed similar analyses using asynchronous cell populations and in agreement we observed that mitotic entry was blocked (data not shown).

**Figure 5:**
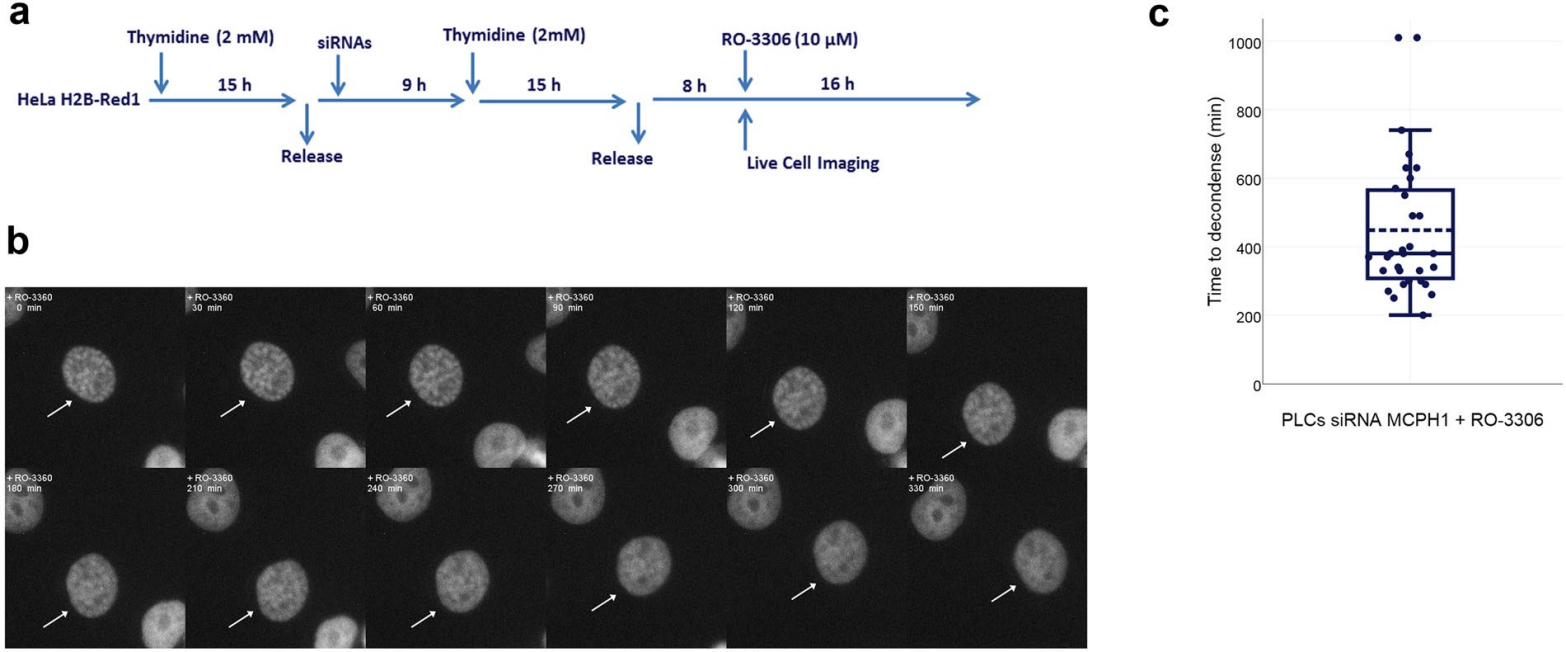
Timing of PLC decondensation after incubation with the Cdk1 inhibitor RO-3360 in HeLa-H2B/Red1 cells. (**a**) Short description of the experimental procedure. RO-3306 was added 8 h after release from the second thymidine block to coincide with the occurrence of PLCs during G2 in the siRNA treated cells. (**b**) Selected frames showing the decondensation of a PLC (pointed by arrows) after incubation with RO-3360. Time after RO-3360 addition is indicated in minutes. (**c**) Combined dot- and box-plot showing the time (in minutes) that PLCs required to completely decondense their chromosomes after adding RO-3360. The broken line indicates the mean value. 30 PLCs were monitored.

We next asked if the maintenance of chromosome condensation in PLCs requires Cdk1 activity. Following MCPH1 depletion and cell synchronization, as described above, we added RO-3306 8 hours after release from the thymidine block, at the time when we observed the peak of PLCs in G2 (based on the data in Fig. 2) (experimental outline detailed in Fig. 5a). This revealed that RO-3306 induced a progressive reduction in condensation in most PLCs until these cells had completely decondensed their chromatin (Fig. 5b and video 5). The time required to completely decondense the chromosomes after adding RO-3306 was 409 (±142) minutes (Fig. 5c). These data are in agreement with the PLC dynamics observed in MCPH1 patient cells after Cdk1 inhibition and suggest that PLCs decondense their chromosomes through a slow but progressive process if Cdk1 is inactivated.

**Figure 6:**
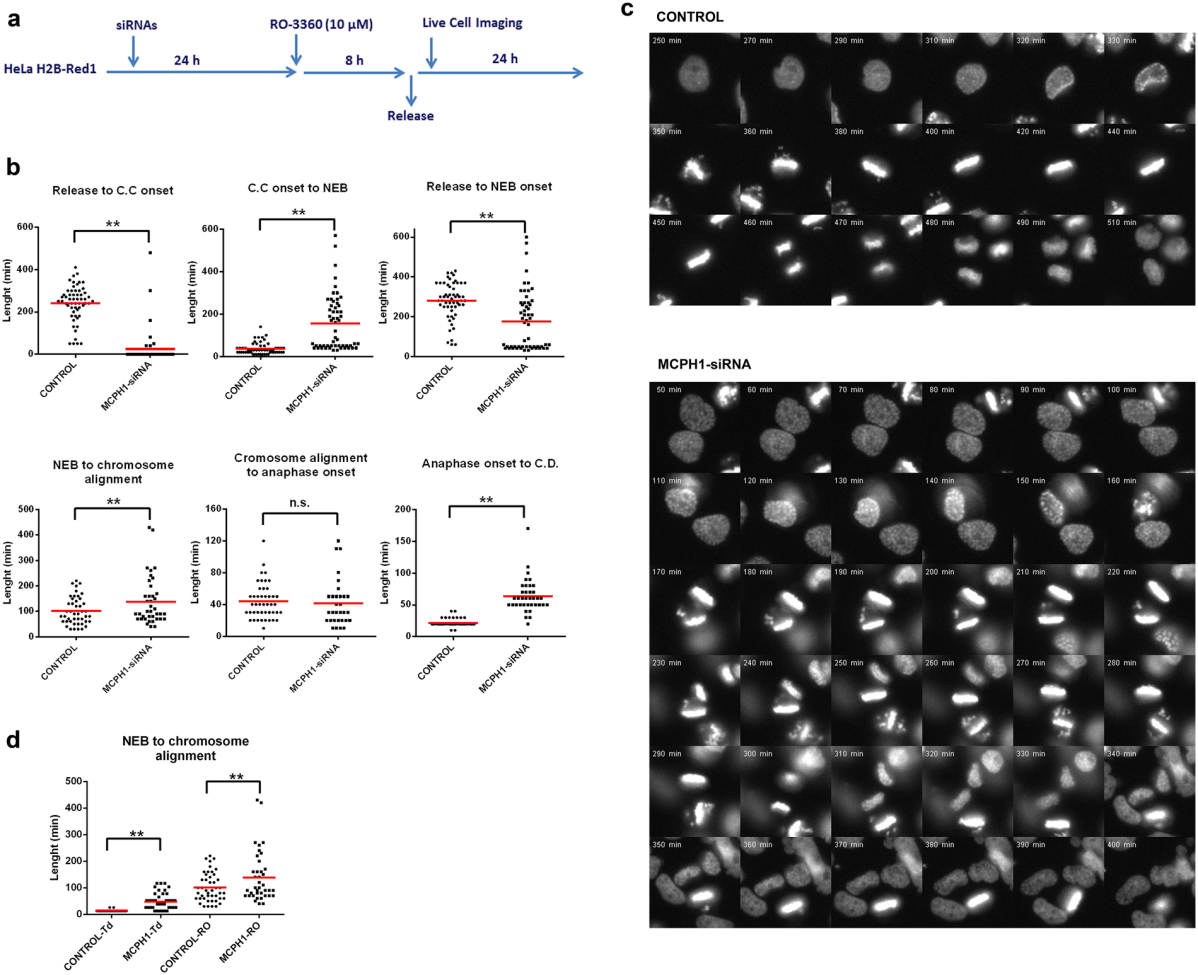
Analyses of mitosis progression by live-cell microscopy in cells depleted of MCPH1 by siRNAs and incubated with the Cdk1 inhibitor RO-3360. (**a**) A brief description of the experimental procedure. HeLa H2B/Red1 cells were arrested in G2 by incubation with RO-3360 for 8 h and then released into normal fresh medium without the inhibitor. MCPH1 depletion was achieved by transfection with siRNAs during the previous 24 hours. Time-lapse recording started 30 minutes after the release from the RO-3360 incubation. Images were analyzed and processed using Image J software. (**b**) Dot-plots showing the time interval between different key mitotic events in minutes for untreated and MCPH1 depleted cells. The red line indicates the mean value. CC = chromosome condensation; NEB = nuclear envelope breakdown; CD = chromosome decondensation. At least 40 cells were analyzed in each case. Chromosome segregation and further decondensation were not analyzed in separate as both occur nearly simultaneously in control cells. Statistical comparisons for the mean and median data were done by T-student and Wilcoxon (W) tests respectively. **p < 0.01; N.S. not significant. (**c**) Selected frames showing the mitotic progression of representative HeLa cells from both control and MCPH1-depleted samples. Time from RO-3360 release is indicated in minutes. (**d**) Pairwise comparison of prometaphase timing (duration) in HeLa-H2B/Red1 cells after release from either double block with thymidine (Td) or RO-3360 incubation (RO).

Lastly, we asked if RO-3306-induced G2 arrest was reversible in MCPH1 depleted cells. HeLa-H2B-Red1 cells were incubated with the Cdk1 inhibitor for 8 hours, then cells were washed with normal medium and monitored by time-lapse microscopy. MCPH1 depletion was achieved by siRNA transfection 24 hours before the incubation with RO-3306 (Fig. 6a). Strikingly, PLCs were observed soon after the removal of RO-3306 in cells depleted of MCPH1 (25 ± 85 min from removal) and these cells remained in that state with prematurely condensed chromatin for 118 (±95) minutes before NEB (Fig. 6b and c, videos 6 and 7). In contrast, chromosome condensation was initiated later in control cells (241 ± 84 minutes from inhibitor removal) and was visible only 36 (±27) minutes before NEB. Therefore, the onset of NEB occurred with significant delay in control cells (280 ± 93 minutes, from inhibitor removal) compared with PLCs (133 ± 130 minutes, from inhibitor removal). After NEB, MCPH1 depleted cells required more time to align all their chromosomes at the metaphase plate (101 ± 54 minutes in control, 149 ± 101 minutes in MCPH1). However, once aligned, anaphase onset occurred with similar timing in both samples (44 ± 22 minutes in control, 38 ± 28 minutes in MCPH1). The amount of time that cells required to exit from mitosis and completely decondense their chromosomes was increased in MCPH1 depleted cells. Control cells required 22 (±5) minutes to complete this step while MCPH1 depleted cells required 64 (±27) minutes. Together these experiments have provided evidence that inhibition of Cdk1 by RO-3306 induces G2-arrest and chromosome decondensation in MCPH1 depleted cells. After removal of RO-3306, MCPH1 depleted cells condensed their chromosomes prematurely and progressed more quickly into mitosis than controls. The premature onset of NEB after recovery from Cdk1 inhibition could be a consequence of a faster rate of Cdk1 reactivation in MCPH1 depleted cells. Importantly, the mitotic phenotypes observed without prior Cdk1 inhibition were also recapitulated in these experiments: prolonged condensation after anaphase and inefficient prometaphase chromosome alignment.

## Discussion

Several studies of MCPH1 function using models including MCPH1 patient cells, depletion of MCPH1 via RNAi and MCPH1 knock-out animals have consistently revealed a key role as a regulator of chromosome condensation. In this paper we have tracked the dynamics of chromosome condensation and cell cycle progression in MCPH1 deficient cells during unperturbed cell division cycles. Our results clearly showed that, in addition to coupling chromosome condensation to other cell division processes, MCPH1 is also required for efficient chromosome alignment during prometaphase.

Our live cell analyses using synchronized cells showed that MCPH1 deficient cells progress through G2 phase and start mitosis on schedule, judged by the timing of NEB. This result was corroborated by our finding that most cells that had undergone premature chromosome condensation (PLCs) in MCPH1 patients were not positive for mitotic markers - nuclear H3PS10 and H3PS28 - and had low levels of nuclear cyclin B, in agreement with previous studies^14,16^. Moreover, we also observed that mitotic cells accumulate at similar rates in control and MCPH1 patient cells during time-course analyses. Together the data demonstrate that PLCs are either G2 or G1 cells that arise as a consequence of chromosome condensation being uncoupled from normal cell cycle progression. Since the abnormal condensation pattern is Cdk1 dependent (see below), it is likely to be a regulatory process rather than an aberrant change in chromatin structure. Despite this, the molecular pathways regulating the G2/M transition remain intact in MCPH1 PLCs^21^.

It has been proposed that premature onset of chromosome condensation depends on premature activation of Cdk1 during G2 in MCPH1 syndrome^16,17^. Here we revealed by live-cell imaging that inhibition of Cdk1 activity in HeLa cells depleted of MCPH1 induces G2 arrest in the absence of chromosome condensation, a behavior similar to that observed in control cells. Moreover, PLCs require continued Cdk1 activity for the maintenance of chromosome condensation. Both G2 arrest and decondensation of PLCs was also observed in MCPH1 patient cells after prolonged Cdk1 inhibition. Interestingly, chromosome condensation starts immediately after release from prolonged Cdk1 inhibition in MCPH1 deficient cells, while in control cells it occurs significantly later. Taken together, our results clearly demonstrate that active Cdk1 is required for the premature onset of chromosome condensation and the maintenance of the condensed state in cells lacking MCPH1 function.

The exact role of MCPH1 in Cdk1 regulation and how that modulates chromosome condensation during an unperturbed cell cycle is currently unknown, and seems to occur independently of the ATR pathway^16,20^. Full activation of Cdk1, the key event triggering cell entry into mitosis, relies on a redundant network of interactions that function in feedback loops to guarantee proper coordination of the different mitotic events^22^. Although the bulk of Cdk1 becomes activated at the G2/M border, lower levels of active Cdk1 are detected early in G2^23^. In this context, our data could indicate that MCPH1 prevents, through an unknown pathway, the induction of chromosome condensation by regulating or sustaining the activation threshold of Cdk1 with regard to chromosome condensation. Alternatively, it has not been ruled out that MCPH1 and Cdk1 regulate chromosome condensation independently since a direct relation between both during this process has not been established.

Mechanistically, premature chromosome condensation in cells lacking MCPH1 results from premature loading of condensin II onto chromatin^14,15^. MCPH1 directly inhibits condensin II loading though competitive binding to the same chromatin domains, thus preventing condensation^15^. Condensin II is phosphorylated by Cdk1 at the T1415 residue of CAP-D3 subunit in early mitosis, which is critical for sustaining further Plk1 phosphorylation and, thus, full activation of condensin II^24^. In this scenario it is reasonable to infer that MCPH1 controls condensin II function directly through physical inhibition of its loading onto chromatin and indirectly by temporal control of condensin II activation mediated by Cdk1-Plk1 phosphorylation.

In this study we revealed a novel function for MCPH1 during mitosis. Live-cell analyses showed that MCPH1 deficient cells require more time to align all the chromosomes at the metaphase plate compared with controls. While most chromosomes appeared to have aligned within ten minutes after NEB, a small number of unaligned chromosomes usually persisted and required more time to complete biorientation. When prometaphase and metaphase progression was analyzed in detail in MCPH1 patient cells a similar alteration was noticed. Consequently, the length of prometaphase is extended and the total length of mitosis is increased in MCPH1 deficient cells. This alteration, not previously reported, indicates a function for MCPH1 in chromosome alignment during mitosis. In previous studies it was proposed that MCPH1 deficiency induces premature entry of cells into mitosis. This conclusion was mainly supported by the increased frequency of H3PS10 positive cells observed in either siRNA-MCPH1 treated cells or patient cell cultures^17,18^ (our present study, Fig. 1 and Supplementary Figure 1). However, the data presented here reveal that the increased mitotic index can be explained by the extended prometaphase duration in cells lacking MCPH1 function, rather than by an accelerated mitotic entry. One study reported elongated mitosis in cells depleted of MCPH1, based on time-lapse microscopy, but the dynamics of chromosome condensation were not described, and no abnormalities in chromosome alignment were reported^18^.

Immunofluorescence analyses in neuroprogenitor cells from a Mcph1 mouse model reported increased frequencies of bipolar spindles with unaligned chromosomes^17^. The occurrence of these errors was explained by uncoupling of centrosome maturation from mitotic processes. We here showed *in vivo* that human cells depleted of MCPH1 require more time to achieve full alignment of chromosomes at the metaphase plate. Taking together our results and previous ones^17^, it seems evident that the process of chromosome alignment during prometaphase is compromised by MCPH1 deficiency. Moreover, the resulting mitotic delay may add information to the current discussions about the pathogenic mechanisms of MCPH1 primary microcephaly^25^. Even a subtle increase in the duration of mitosis could deeply impact the final production of neurons during neurogenesis, while in other tissues types it would not induce noticeable alterations.

Currently we do not understand the molecular basis for the deficiency during prometaphase chromosome alignment that is observed. Minor alterations in the dynamics of kinetochore-microtubule attachments and/or chromosome movements towards the spindle equator offer possible explanations. The disturbed maturation of the centrosome reported in Mcph1 −/− mouse cells could be one critical underlying factor^17^. Besides that, it is important to note that mitotic chromosome structure is disturbed by a lack of functional MCPH1^10^ (Supplementary Figure 2). Apart from a hypercondensation the resolution of sister chromatids is delayed; a critical process to assure faithful chromosome segregation. Notably, a recent study establishes that decatenation failure is a novel pathogenic mechanism for microcephaly in condensin II-mutated patients^26^. Given the known interaction of condensin II with MCPH1^14,15^, the occurrence of unresolved sister chromatids^10^ and the extended prometaphase reported here for human cells lacking MCPH1 function, it is interesting to consider that altered decatenation activity during mitosis directly contributes to the occurrence of MCPH1 primary microcephaly. The slight increase in the number of segregation errors observed during anaphase provides additional support to this scenario, as incomplete decatenation is one of the main mechanisms underlying lagging or bridged chromosomes^27–29^.

## Material and Methods

### Cell cultures and treatments

We have used the next standard human cell lines: HeLa modified (H2B-Red1 tagged; H2B-GFP tagged), Hct-116 modified (H2B-GFP tagged) and U2OS. Also we have employed lymphoblast cell lines (LCLs, non-transformed EBV immortalized) from one MCPH1 patient (S25X mutation) and one healthy control subject^6,7^. Adherent cell lines were grown following standard conditions using DMEM medium supplemented with 10% of foetal bovine serum. LCLs were grown under usual conditions in RPMI medium supplemented with 15% foetal bovine serum.

For RNAi treatments cells were transfected with 120 nM siRNA duplexes using Lipofectamine (Invitrogen) at 50% confluency. OptiMEM medium (Invitrogen) was used for cell transfection. RNA oligos were purchased from Qiagen. The sequences of the siRNA duplexes used deplete specifically both major isophorms of MCPH1 mRNA, and were based on a previous study^11^. These validated siRNAs oligos knocked-down the MCPH1 protein levels efficiently [10, 11, 14, Supplementary Figure 3]. Synchronization of cells at G1/S was achieved by a double-thymidine protocol. The inhibitors employed were nocodazole (Sigma-Aldrich, final concentration 1,5 µM) and RO-3306 (Sigma-Aldrich; final concentration 10 µM). Untreated control cells were incubated in all cases with a similar volume of dimethyl sulfoxide.

### Live-cell microscopy

Cells were plated onto 35 mm tissue culture dishes fitted with glass cover-slips (MatTek Cultureware). siRNA transfection and thymidine synchrony was performed as described in the results section, except that upon release from the second thymidine arrest or before imaging (when monitoring asynchronous cells) the standard medium containing the thymidine was exchanged for DMEM without phenol red, supplemented with 10% FBS, penicillin/streptomycin and 200 mM Trolox (Calbiochem). The dishes were transferred to a microscope humidified stage incubator containing 5% CO2 at 37 °C. Cells were filmed with three to five z sections using a Nikon Biostation IM microscope fitted with 20x and 40x/0.8 n.a. objectives and coupled with Biostation IM software. Images were stacked and processed using Image J software. Timing data were obtained after visual inspection of a minimum of 40 cells. Statistical comparisons were done using Statgraphics software.

### FACS

Flow cytometry analyses were done using lymphoblast cell cultures in log-phase. One million cells approximately were recovered, washed in PBS and fixed in ice-cold Ethanol 70 overnight. Phospho-histone H3 positive cells were detected with a rabbit anti-histone H3PS10 antibody (Abcam) at a dilution of 1/250, and a donkey anti-mouse IgG FITC-conjugated secondary antibody (Santa Cruz). Propidium iodide was used as a counterstain for DNA content. Fluorescence detection was performed using an analytical flow cytometer (LSR Fortessa, BD Bioscience) equipped with BD FACSDiva™ software for data acquisition. Quantitative cell cycle analysis was done with Flowing Software v.2.5.1.

### Cytogenetic analyses

Cytogenetic preparations following standard protocols were obtained in parallel from the same log-phase cell cultures analyzed by FACS. Chromosome preparations were fixed using Carnoy’s solution (methanol/glacial acetic acid, 3:1), stained with Giemsa (10%) and finally visualized at microscope. The fraction of “prophase-like cells” (PLCs) and metaphases was determined after counting 1000 nuclei from coded slides at microscope. For the detailed analyses of the process of chromosome alignment in prometaphase and metaphase cells we employed cytogenetic preparations obtained following a protocol that preserves the organization of chromosomes on the mitotic spindle^12^. Microscopy images were captured with a CCD camera (Olympus DP70) coupled to a microscope (Olympus BX51) and finally managed with ImageJ software.

### Immunofluorescence

SV-40 transformed fibroblasts from one MCPH1 patient (mutation T143nfsX5) and one health control were analyzed as previously described^11^. Cells growing on glass coverslips were fixed with 4% paraformaldehyde in PBS (pH 7.4) for 15 min at room temperature and permeabilized with ice-cold methanol for 30 min on ice. Cells were incubated with PBS containing 20% FBS as a blocking agent for 30 min and then with the indicated antibodies for approximately 1 h at room temperature. After being washed three times with PBS, cells were incubated with the respective secondary antibodies conjugated with fluorescence dyes. After counterstaining with DAPI, coverslips were mounted with VECTASHIELD and examined with a Zeiss Axioskop microscope equipped with a cooled charge-coupled device (CCD) camera. Grayscale images were pseudocolored and merged using ImageJ. Primary antibodies used were mouse anti-Cyclin B1 (Abcam), rabbit anti-H3PS10 (Cell Signaling), rabbit anti-H3PS28 (Upstate).

### Immunoblots

For immunoblotting, cells were lysed in 1x SDS sample buffer containing 60 mM Tris-HCl [pH 6.8], 1% SDS, 10% glycerol, 0.01% bromophenol blue, and 0.1 M DTT. 1 × 10^5^ cells were suspended in 100 μl of lysis buffer, sonicated and boiled for 2 min. Proteins were resolved by SDS-PAGE and transferred to Hybond-P PVDF membranes (Amersham). The membrane was blocked with 5% (w/v) dry milk in TBS-T (20 mM Tris-HCl [pH 7.5], 150 mM NaCl, 0.05% Tween 20). Incubation with primary antibodies was performed in TBS-T containing 1% BSA and 0.05% sodium azide for 1 hour at room temperature. Alpha-tubulin (Sigma) was used as loading control. Blots were developed by enhanced chemiluminescence detection system (Amersham). The antibody against human MCPH1 was kindly provided Dr. Tatsuya Hirano (RIKEN, Japan).

### Data Availability

All data generated or analyzed during this study are included in this published article (and its Supplementary Information files).

## Electronic supplementary material

Supplementary figures

Supplementary Video 1

Supplementary Video 2

Supplementary Video 3

Supplementary Video 4

Supplementary Video 5

Supplementary Video 6

Supplementary Video 7

Supplementary Video 8
