# Supplementary material for: MCPH1, mutated in primary microcephaly, is required for efficient chromosome alignment during mitosis
Source: Sci Rep. 2017 Oct 12;7:13019. doi: 10.1038/s41598-017-12793-7 (PMC5638862; doi:10.1038/s41598-017-12793-7)
Supplement: Supplementary file 1 — Supplementary figures [file 41598_2017_12793_MOESM1_ESM.pdf]

Supplementary information: Arroyo *et al*.

MCPH1, mutated in primary microcephaly, is required for efficient chromosome alignment during mitosis

M. Arroyo[1], R. Kuriyama[2], M. Trimborn[3], D. Keifenheim[2], A. Cañuelo[1], A. Sánchez[1], D. J. Clarke[2], J.A. Marchal[1*]

* Corresponding author: Dr. Juan Alberto Marchal Ortega, Departamento de Biología Experimental; Facultad de Ciencias Experimentales, Universidad de Jaén, Paraje Las Lagunillas s/n; Room B3-304, E-23071 Jaén (Spain), Telephone: 0034-953213361; Fax: 0034-953211875, e-mail: jamaor@ujaen.es

Supplementary Figure S1

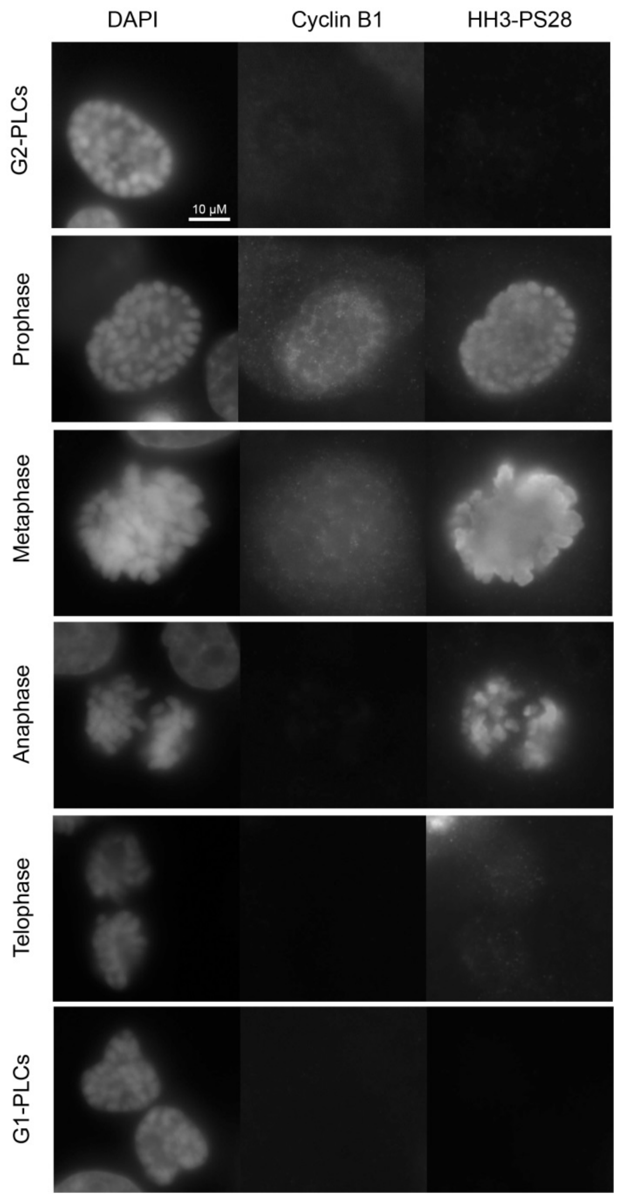

Supplementary figure S1. Immunolocalization using antibodies against Cyclin B and Histone H3-PS28 proteins in proliferating cells from one MCPH1 patient.

Supplementary Figure S2

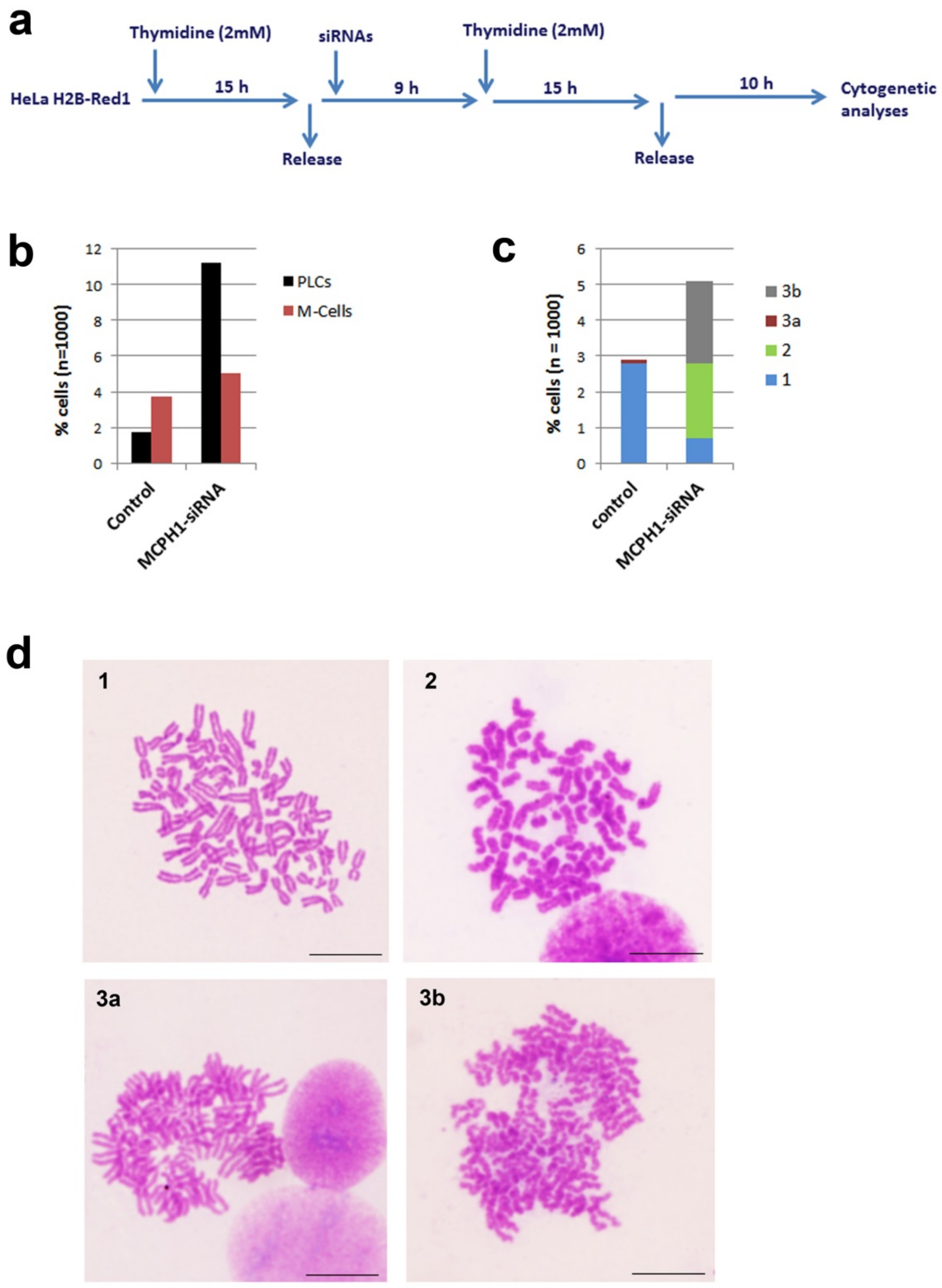

Supplementary figure S2. Cytogenetic analyses of the occurrence of PLCs and chromosome morphology in HeLa H2B-Red1 cells depleted of MCPH1 function by siRNAs. a) A brief diagram of the experimental protocol used. b) Fraction of PLCs and mitotic cells determined by visual inspection by microscopy, n=1000. c) Quantitative analyses of the chromosome morphology observed within the mitotic cells from B. d) Representative images of the main chromosomal morphologies observed in our analyses. As expected, in MCPH1-siRNA treated cells chromosomes with a wavy hypercoiled appearance and unresolved sister chromatids (type 2) or with premature centromeric division (type 3b) were frequently observed. However, in control cells chromosomes present straight, well resolved chromatids that remain connected at the centromeres in most cases (type 1), being premature centromeric division barely observed (type 3a).

Supplementary Figure S3

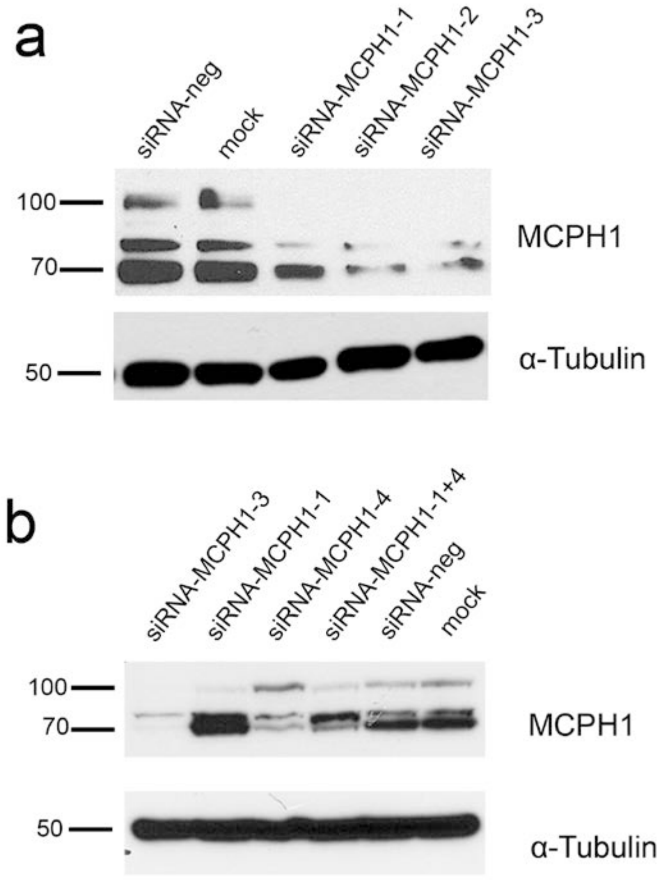

Supplementary figure S3. Immunoblots with an anti-MCPH1 antibody after transfection of HeLa (a) and U2OS (b) cells with different siRNAs showing efficient depletion of MCPH1 protein. Oligos siRNA-2 and siRNA-3 downregulate both described isoforms, full-lenght (93 kDa) and Δ9-14 (70 kDa) (Gavvovvidis et al. 2012). Oligos siRNA-1 and siRNA-4, targeting full-length and Δ9-14 isoforms respectively, specifically downregulate each of them. Non-targeting siRNA (siRNA-neg) or mock-transfected (mock) controls showed no downregulation. Alpha-tubulin served as a loading control. The rabbit polyclonal antiserum against MCPH1 was kindly furnished by Dr. Tatsuya Hirano (RIKEN, Japan).

Supplementary Figure S4

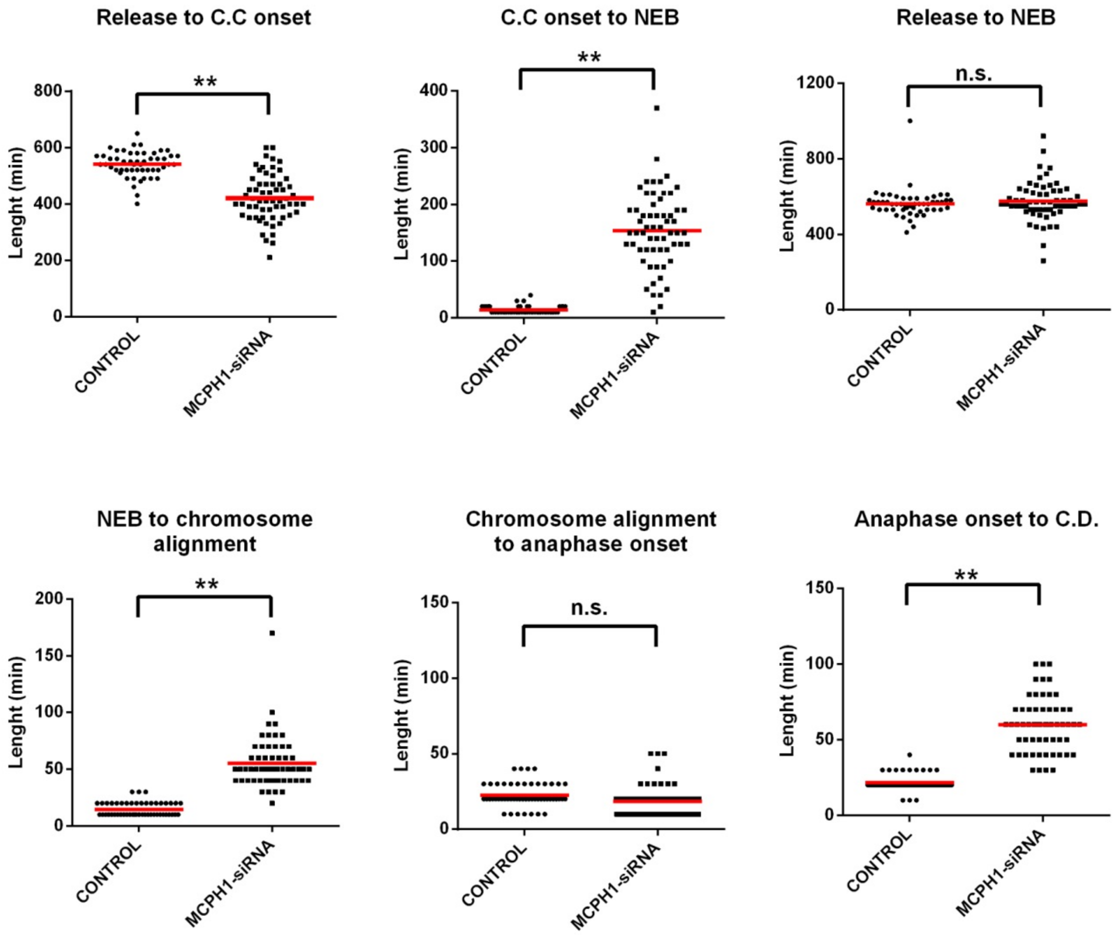

Supplementary figure S4. Dot-plots showing the time interval between different key mitotic events in HeLa-Red1 cells either mock treated or depleted of MCPH1 function with a second non-overlapping siRNA (oligo siRNA-MCPH1-2). Cells were processed as described in Figure 2. The red line indicates the mean value. C.C. = chromosome condensation; NEB = nuclear envelope breakdown; C.D. = chromosome decondensation. More than 50 cells were analyzed in each case. Statistical comparisons for the mean and median data were done by T-student and Wilcoxon (W) tests respectively. ** p<0.01; N.S. not significant.

Supplementary Figure S5

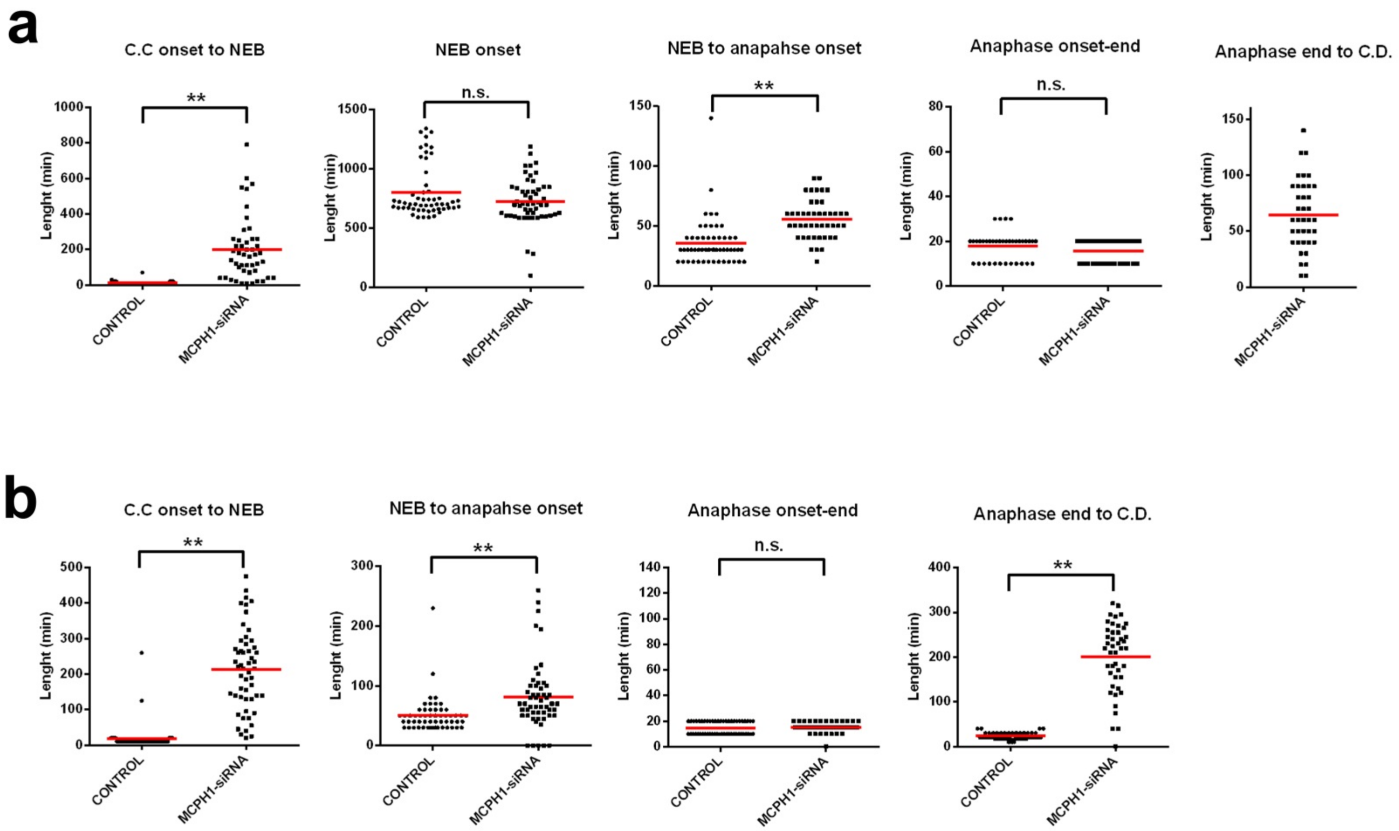

Supplementary figure S5. Dot-plots showing the time interval between different key mitotic events in minutes in Hct-116 (a) and HeLa-GFP (b) cells either mock treated or depleted of MCPH1 function by siRNAs. The red line indicates the mean value. C.C. = chromosome condensation; NEB = nuclear envelope breakdown; C.D. = chromosome decondensation. At least 50 cells were analyzed in each case. In Hct-116 mock control cells chromosome segregation and further decondensation were not analyzed in separate as both occur nearly simultaneously. Statistical comparisons for the mean and median data were done by T-student and Wilcoxon (W) tests respectively. ** p<0.01; N.S. not significant.

Supplementary Figure S6

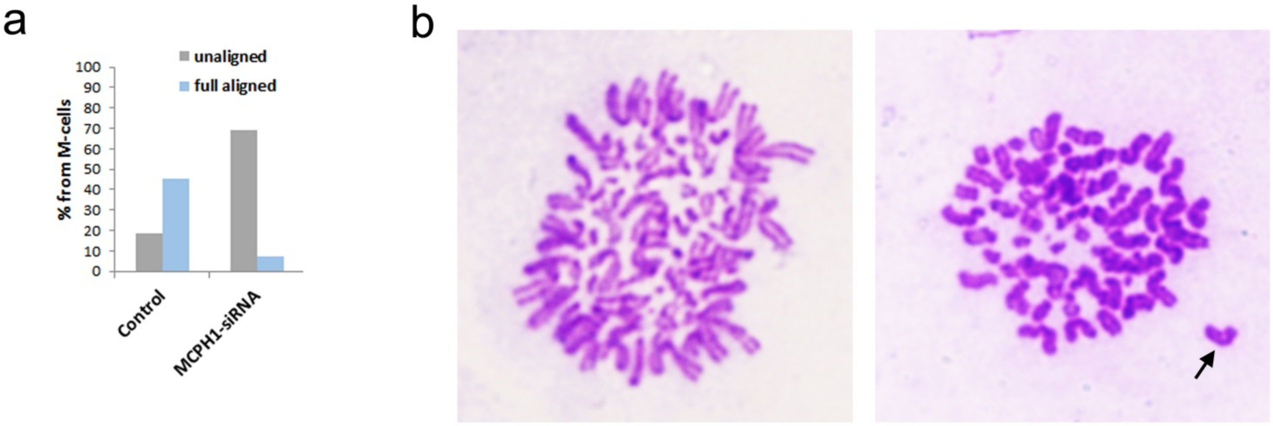

Supplementary figure S6. (a) Fraction of U2OS mitotic cells showing either all chromosomes organized into the metaphase plate or some unaligned chromosomes. Unsynchronized cells either mock treated or depleted of MCPH1 function by siRNAs were used. Analyses were performed by microscopic inspection of cytogenetic preparations obtained following a protocol that preserves the organization of chromosomes on the mitotic spindle [12]. 100 mitotic cells were counted and classified in each case. (b) Representative images of mitotic cells classified in A as aligned (left picture) or displaying some unaligned chromosomes (pointed by arrow, right picture). In MCPH1-siRNA treated cells, this particular phenotype was frequently observed and resemble to the observed by live-cell in HeLa cells (Figure 3C) and in patient cells (Figure 3E).

Supplementary Figure S7

a

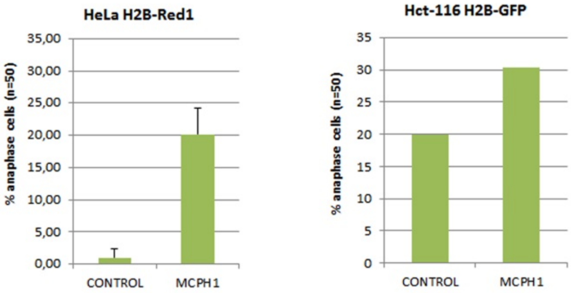

b

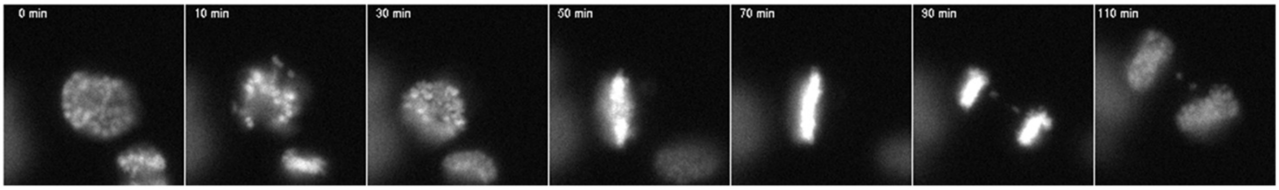

Supplementary figure S7. a) Percent of cells showing bridge or lagging errors during anaphase in either control or MCPH1 depleted cells. Two different cell lines were analyzed. For HeLa cells data from experiments employing either thymidine or RO-3306 synchronization protocols are shown. b) Live-imaging stack from HeLa H2B-Red1 cells depleted of MCPH1 function showing an example of lagging error during anaphase. Time (in minutes) from nuclear envelope breakdown (first frame) is indicated.

Supplementary videos information

Video 1: video showing HeLa H2B-Red1 control cells recorded after release from second thymidine arrest. Time from release (in minutes) is indicated.

Video 2: video showing HeLa H2B-Red1 MCPH1-siRNA treated cells recorded after release from second thymidine arrest. Time from release (in minutes) is indicated.

Video 3: video of HeLa H2B-Red1 control cells released from second thymidine arrest were immediately incubated with RO-3306 and recorded. Time from RO-3306 adding (in minutes) is indicated.

Video 4: video of HeLa H2B-Red1 MCPH1-siRNA treated cells released from second thymidine arrest were immediately incubated with RO-3306 and recorded. Time from RO-3306 adding (in minutes) is indicated.

Video 5: video of a representative PLC showing progressive decondensation after RO-3306 adding. Time from RO-3306 adding (in minutes) is indicated.

Video 6: video of HeLa H2B-Red1 control cells incubated with RO-3306 during 8 hours were released into normal medium and recorded. Time from RO-3306 release (in minutes) is indicated.

Video 7: video of HeLa H2B-Red1 MCPH1-siRNA treated cells incubated with RO-3306 during 8 hours were released into normal medium and recorded. Time from RO-3306 release (in minutes) is indicated.

Video 8: video of HeLa H2B-GFP MCPH1-siRNA treated cells recorded after release from second thymidine arrest. Time from release (in minutes) is indicated. PLCs before NEB and after chromosome segregation are clearly observed.
