# Supplementary figures and images for: MCPH1, mutated in primary microcephaly, is required for efficient chromosome alignment during mitosis
Source: Sci Rep. 2017 Oct 12;7:13019. doi: 10.1038/s41598-017-12793-7 (PMC5638862; doi:10.1038/s41598-017-12793-7)

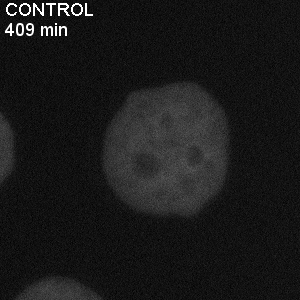

Supplement: Supplementary file 2 — Supplementary Video 1 [file 41598_2017_12793_MOESM2_ESM.gif]

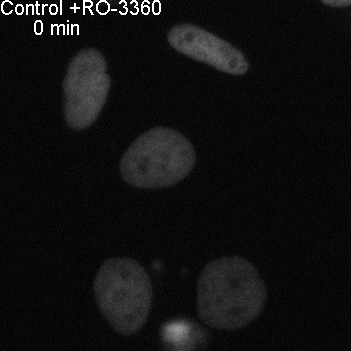

Supplement: Supplementary file 4 — Supplementary Video 3 [file 41598_2017_12793_MOESM4_ESM.gif]

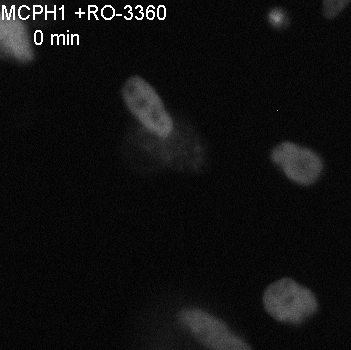

Supplement: Supplementary file 5 — Supplementary Video 4 [file 41598_2017_12793_MOESM5_ESM.gif]

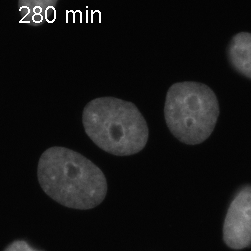

Supplement: Supplementary file 9 — Supplementary Video 8 [file 41598_2017_12793_MOESM9_ESM.gif]
